# Transgenerational effect alters the interspecific competition between two dominant species in a temperate steppe

**DOI:** 10.1002/ece3.7066

**Published:** 2021-01-10

**Authors:** Yang Li, Longyu Hou, Liuyi Yang, Ming Yue

**Affiliations:** ^1^ Xi’an Botanical Garden of Shaanxi Province Institute of Botany of Shaanxi Province Xi’an China; ^2^ Shaanxi Engineering Research Centre for Conservation and Utilization of Botanical Resources Xi’an China; ^3^ State Key Laboratory of Vegetation and Environmental Change Institute of Botany Chinese Academy of Sciences Beijing China; ^4^ Key Laboratory of Resource Biology and Biotechnology in Western China Northwest University Xi’an China

**Keywords:** competition, global change, maternal effect, nitrogen deposition, water addition

## Abstract

One of the key aims of global change studies is to predict more accurately how plant community composition responds to future environmental changes. Although interspecific relationship is one of the most important forces structuring plant communities, it remains a challenge to integrate long‐term consequences at the plant community level. As an increasing number of studies have shown that maternal environment affects offspring phenotypic plasticity as a response to global environment change through transgenerational effects, we speculated that the transgenerational effect would influence offspring competitive relationships. We conducted a 10‐year field experiment and a greenhouse experiment in a temperate grassland in an Inner Mongolian grassland to examine the effects of maternal and immediate nitrogen addition (N) and increased precipitation (Pr) on offspring growth and the interspecific relationship between the two dominant species, *Stipa krylovii* and *Artemisia frigida*. According to our results, *Stipa kryloii* suppressed *A. frigida* growth and population development when they grew in mixture, although immediate N and Pr stimulated *S. kryloii* and *A. frigida* growth simultaneously. Maternal N and Pr declined *S. krylovii* dominance and decreased *A. frigida* competitive suppression to some extent. The transgenerational effect should further facilitate the coexistence of the two species under scenarios of increased nitrogen input and precipitation. If we predicted these species' interspecific relationships based only on immediate environmental effects, we would overestimate *S. krylovii*'s competitive advantage and population development, and underestimate competitive outcome and population development of *A. frigida*. In conclusion, our results demonstrated that the transgenerational effect of maternal environment on offspring interspecific competition must be considered when evaluating population dynamics and community composition under the global change scenario.

## Graphical abstract

Maternal environments could affect offspring performance and offspring competitive ability. Transgenerational effects should further facilitate coexistence under scenarios of nitrogen addition and increased precipitation. Transgenerational effects of maternal environment on offspring interspecific competition must be taken into account for predicting population dynamic and community composition under global change scenarios.

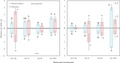

## 1 INTRODUCTION

The global environment is undergoing rapid change, owing to a variety of natural and anthropogenic events (MEA, 2005). Concurrent with climate warming, precipitation is projected to increase in midlatitude terrestrial ecosystems (Donat et al., 2016; IPCC, 2013; Knapp et al., 2015). As fertilization or atmospheric deposition, nitrogen input into terrestrial ecosystems has been increased from 34 to 100 Tg N/year, and it will reach 200 Tg N/year by 2050 (Galloway et al., 2008). One of the central goals of global change studies is to accurately predict how plant diversity and community composition respond to future environmental changes (Bellard et al., 2012; Dawson et al., 2011; Gilman et al., 2010; Moritz & Agudo, 2013; Zhang et al., 2019). It is generally agreed that interspecific competition is among the most important forces structuring plant communities (Aerts, 1999; Niu & Wan, 2008) and is commonly environment‐dependent (Gilman et al., 2010). Studies on transgenerational effects have shown that maternal environment affects offspring performance (Franks et al., 2007; Galloway, 2001; Huxman et al., 2001; Miska & Ferguson‐Smith, 2016). However, little is known about how the maternal environment influences interspecific competition through the transgenerational effect. Because offspring may exhibit different plasticities as a response to environmental changes from that of the maternal plant through transgenerational effect (Lau et al., 2008; Li et al., 2011), the interspecific relationship of offspring plants in response to environmental change may also be different from that of the maternal plant. Therefore, to accurately evaluate future response of community composition to environmental change, it is important to ascertain how maternal nitrogen addition or/and increased precipitation affect interspecific competition among maternal plants or among offspring plants.

The transgenerational effect could alter interspecific competition by altering offspring performance and plasticity (Miska & Ferguson‐Smith, 2016; Rasanen & Kruuk, 2007). As sessile organisms with limited escape ability (Holeski et al., 2012), plants have to adapt or they become extinct as a response to environmental changes (Yin et al., 2019). According to studies on transgenerational effects, plants not only have enormous potential for phenotypic plasticity to immediate environmental change (Neytcheva & Aarssen, 2008), but they also transfer plasticity to their offspring (Franks et al., 2007; Galloway, 2001; Huxman et al., 2001; Li et al., 2011, 2017, 2019). The transgenerational effect of maternal environment and plasticity effect of the immediate environment both influence offspring performance (Auge et al., 2017), although the direction and magnitude of the two effects may be different (Lau et al., 2008; Li et al., 2011). Consequently, competitive ability, which is calculated for each trait, may be simultaneously affected by maternal environment and immediate environment. It is essential to quantify competitive ability or outcome not only through the plasticity effect but also through the transgenerational effect because being a good competitor or surviving under competition may occur via pluralistic approaches in the parental generation or in filial generations (Aarssen & Keogh, 2002; Aschehoug et al., 2016; Hart et al., 2018; MacDougall & Turkington, 2004; Wang et al., 2010). Therefore, competitive outcome in response to immediate environment only, which has been tested in many previous studies, is insufficient to represent long‐term competitive outcomes and consequent community composition (Freckleton et al., 2009; Hart et al., 2018; Laughlin, 2014; Tylianakis et al., 2008; Yin et al., 2019).

Theoretical (Ezard et al., 2014; Galloway & Etterson, 2007) and experimental (Burgess & Marshall, 2011) studies have shown that the transgenerational effect on offspring is adaptive when the environment is predictable (Uller, 2008). In addition, the environment could be anticipated because environmental change is correlated under the global change scenario. If offspring traits can be affected by transgenerational effects, considering that the transgenerational effect of each species in a community is adaptive, how does interspecific competition change and how does community composition change as a consequence? Relatively, little is known about how the maternal environment influences interspecific competition during the adaptation of each species to a changing environment. Therefore, quantifying the effect of the maternal environmental effect on interspecific competition is crucial for accurately evaluating the future response of community composition to global changes (Auge et al., 2017).

As an important biome, grasslands occupy 36% of the terrestrial land cover (Sala, 2001). Quantifying the magnitude of the responses of grass community composition to nitrogen and rainfall alterations is crucial for accurately evaluating the global response of plant productivity and diversity to future climate scenarios (Song et al., 2019). To experimentally elucidate whether the maternal environment influences offspring interspecific competition through transgenerational effect, a field and a greenhouse experiment were conducted in a typical steppe in Inner Mongolian grassland. The field experiment was part of a multifactor field experiment initiated in April 2005 (Li et al., 2011; Yang et al., 2012). Increased precipitation and N addition have consistently been documented to profoundly impact community composition and biodiversity in this steppe ecosystem (Xu et al., 2018; Yang et al., 2011, 2012). Moreover, pioneer studies at the site have demonstrated that maternal‐increased precipitation and nitrogen environments play important roles in offspring performance through transgenerational effects (Li et al., 2011, 2017, 2019). Despite the growing evidence on rapid adaptive evolution as a response to environmental changes in the plant population, the consequences of such rapid evolution on plant diversity and community composition remain to be explored (Lavergne et al., 2010). The competitive relationship of dominant species is important for understanding community composition and biodiversity in ecosystems (Fay et al., 2003; Zhang et al., 2017). Thus, *Stipa krylovii* and *Artemisia frigida*, two dominant species that coexist in the study ecosystem (Liu et al., 2016), were selected in our study. The specific objectives of our study were to reveal (a) how immediate nitrogen addition, increased precipitation, and their interaction affect interspecific competition between the two dominant species through phenotypic plasticity; and (b) whether and how maternal nitrogen addition, increased precipitation, and their interaction affect interspecific competition between the two dominant species through the transgenerational effect.

## 2 MATERIALS AND METHODS

### 2.1 Site description

Our study was carried out at a semiarid temperate steppe in Duolun County (42°02ʹN, 116°17′E, 1,324 m a.s.l.), the Inner Mongolia Autonomous Region of China. The long‐term mean annual temperature is 2.4°C, and the mean annual precipitation is 382 mm, of which approximately 80% is concentrated from June to September (Song et al., 2019). According to the FAO classification, the sandy soil at this study site is Haplic Calcisols. The steppe community is dominated by perennials, including *S. krylovii* Roshev., *A. frigida* Willd., *Potentilla acaulis* L., *Potentilla tanacetifolia* Willd., *Cleistogenes squarrosa* (Trin.) King, *Allium bidentatum* Fisc. ex prokh., and *Agropyron cristatum* (L.) Gaertn (Niu & Wan, 2008).

### 2.2 Plant material

*Stipa krylovii* and *A. frigida*, two perennial species in the temperate steppe, were selected for this study. *Stipa krylovii*, a tall bunchgrass, is widespread in arid and semiarid grasslands in Inner Mongolia, China. This species flowers, from the end of July to August, and its seeds are dispersed in September. At the peak of the growing season, it can grow to approximately 50 cm. *Artemisia frigida*, a semishrub, occupies approximately 38% of the total foliar biomass in the steppe ecosystem. This species flowers at the end of August and its seeds are dispersed in October. During the peak of the growing season, its vegetative tiller is usually less than 10 cm and its reproductive tiller can reach 30 cm in height.

### 2.3 Field experimental design and seed collection

The permanent site of the Duolun Global Change Multifactor Experiment (GCME) was established in 2005 in northern China (Li et al., 2011; Yang et al., 2011, 2012). GCME employed a design with N addition manipulated at the plot level and precipitation manipulated at the subplot level. Four pairs of 44 × 28 m plots were established; one plot of each pair was randomly assigned as control (C) and the other as “N addition” (N) treatment. Nitrogen at 10 g N/m^2^ (urea in 2005 and NH_4_NO_3_ in 2006–2015) was applied in the N addition treatments in mid‐July every year. In each control or N addition plot, two 10 × 15 m subplots were set up, of which one was watered in summer and the other was not. From July to August every year, 15 mm of water was added once a week, leading to an annual addition of 120 mm precipitation (approximately 30% of the mean annual precipitation in the study site) in a water addition subplot.

From September to October 2015, seeds of *S. krylovii* and *A. frigida* were collected from GCME. Before seed diffusion, seeds were collected throughout the whole plot or subplot as evenly as possible. For one species, seeds collected from the plots or subplots of each treatment in the field were mixed together. Treatments in GCME were considered to be the maternal environment in our analysis and were named as follows: maternal control environment (M‐CK), maternal nitrogen addition environment (M‐N), maternal‐increased precipitation environment (M‐Pr), and maternal nitrogen addition plus increased precipitation environment (M‐NPr). Thus, in the study, seeds were identified as coming from four maternal environments: M‐CK, M‐N, M‐Pr, and M‐NPr.

### 2.4 Greenhouse experiment

A greenhouse experiment was conducted adjacent to the field site. To be consistent with the environmental conditions of GCME, the greenhouse wall and ceiling were opened on sunny days and closed the greenhouse on rainy days. During the entire experimental period, the temperature of the greenhouse was approximately 22.7°C in the daytime and approximately 12.3°C at night.

After being air‐dried, seeds collected in GCME (treatments: M‐CK, M‐N, M‐Pr, M‐NPr) in 2015 were germinated under the same conditions in late April 2016 in the greenhouse. After approximately 20 days, *S. krylovii* seedlings grew to 10–15 cm and *A. frigida* seedlings grew to 5–7 cm. These seedlings were transplanted into pots (15 cm in diameter and 15 cm in depth) in the middle of May. The pots of the greenhouse experiment were filled with soil from GCME outside the treated plots.

The greenhouse experiment was divided into two parts.

First, in order to observe the effect of maternal environment on interspecific competition, seedlings of two species from the four maternal treatments applied in GCME (M‐CK, M‐N, M‐Pr, M‐NPr) were transplanted into pots. All pots received the same treatment. Each pot was watered with 100 ml to maintain growth every day and received about 600 mm water during the entire growth season (from May to August). These values were higher than the MAP in Duolun County because the sandy soil in small pots and higher temperature in the greenhouse required more water (Li et al., 2019). That is, all the pots used to this point of the experiment were under the same immediate environment; thus, the seedlings from the four maternal environments received the same immediate environment. Furthermore, each pot had two seedlings: two *S. krylovii* seedlings (monoculture of *S. krylovii*), two *A. frigida* seedlings (monoculture of *A. frigida*), or one *S. krylovii* seedling and one *A. frigida* seedling (mixed cultures of the two species). Seedlings in a pot were from the same maternal environment. Six replications were set for each treatment.

Second, in order to observe the effect of the immediate environment on interspecific competition, seedlings from maternal control treatment (M‐CK) in GCME were transplanted into pots in the greenhouse. The seedlings were randomly assigned to four immediate environments crossed with three competition treatments (monoculture of *S. krylovii*, monoculture of *A. frigida*, or mixed cultures of the two species) within a block. The four immediate environments that simulated maternal environments in GCME were applied as control, nitrogen addition, increased precipitation, and N addition and increased precipitation treatments to seedlings in this part of the experiment. To differentiate these treatments from those simulating the maternal environments, we named these four immediate environments as I‐CK, I‐N, I‐Pr, and I‐NPr, respectively. Each pot was watered with 100 ml of water per day to maintain plant growth. Beginning in late May, water was replaced with 100 ml of 0.5 g/L NH_4_NO_3_ solution, which was added to each N addition treatment alone (I‐N) and to the N plus water addition pot (I‐NPr); this was performed 10 times during a 10‐day interval until late August for one year. The precipitation treatment was measured in August with an additional 100 ml of water every day in each I‐Pr or I‐NPr pot. A total of 200 ml of water per day was added to the precipitation‐increased pots in order to make a substantial difference in water availability compared with the ambient watering treatments (100 ml water per day) because of the sandy soil and higher temperature in the greenhouse (Li et al., 2011). That is, the seedlings from the M‐CK treatment received four immediate environments in this part. There were six replications for each treatment.

We harvested plants in late August 2017. At harvest, shoots of one individual were clipped at the soil surface and placed in a paper bag. To identify the shoots, we labeled the roots before clipping the two individuals in monoculture pots. The roots and soil from each pot were placed in plastic mesh bags. The soil was washed from the roots with flowing water. Thereafter, the roots of one individual were placed in a paper bag. Because of the low plant density, sandy soil, and root system morphology differences between the two species, it was feasible to assign roots to individuals and ensure root extraction efficiency (Cahill, 2002; Wang et al., 2010). The dry mass was oven‐dried at 65°C for 48 hr to a constant weight.

### 2.5 Statistical analysis

We used competitive response (CR) to determine competitive ability in our study as CR is highly labile and contingent upon the environment (Goldberg & Fleetwood, 1987; Wang et al., 2010). For each species, CR was measured using the species' biomass when grown with other species (in mixture) divided by its biomass grown with the same species (in monoculture). For each species, seedlings with approximate shoot size, root size, and leaf number before transplanting were chosen. We calculated the CR of each species using the biomass of the species in mixture divided by the biomass in monoculture randomly. As there were six replications for each treatment, there were also six replications for CR. When the CR of *S. krylovii* was calculated, *S. krylovii* was considered the target species and *A. frigida* was the accompanying species. The inverse was also performed. A high CR value indicates a strong ability of the target species to mitigate the cost of competition with the accompanying species (Wang et al., 2010).

Three‐way ANOVAs were used to examine the main and interactive effects of N addition, increased precipitation in maternal or in immediate environments, competition on offspring biomass, and biomass allocation of the two species. Two‐way ANOVAs were used to examine the main and interactive effects of N addition and increased precipitation in the maternal or immediate environment on the CR of the two species. All statistical analyses were conducted using the SAS software (SAS Institute Inc.).

## 3 RESULTS

### 3.1 Immediate environment affected the interspecific relationship of the two species

**Figure 1:**
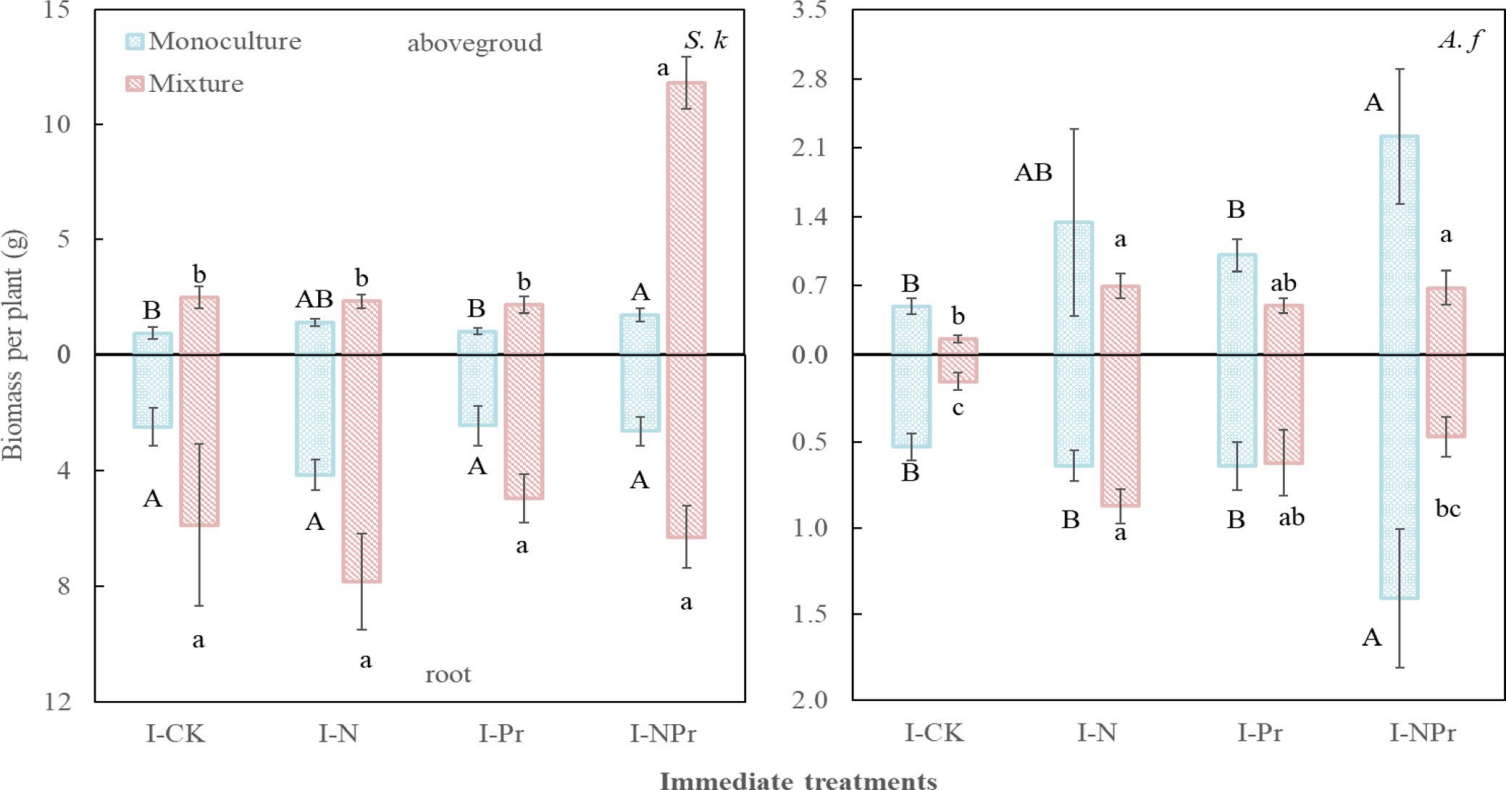
Immediate‐treatment‐induced changes in biomass, aboveground biomass, and belowground biomass of the two species grow in monoculture and in mixture. Values are means ± *SE*, *n* = 6 for each treatment. *S.k*: *Stipa krylovii*, *A.f*: *Artemisia frigida*. I‐CK: control treatment; I‐N: nitrogen addition in greenhouse experiment (immediate N addition environment); I‐Pr: water addition in greenhouse experiment (immediate increased precipitation environment); I‐NPr: nitrogen addition and water addition in combine in greenhouse experiment (immediate N addition plus increased precipitation environment). Different uppercase letters indicate significant differences among treatments for *S. krylovii* or *A. frigida* grown in monoculture; different lowercase letters indicate significant differences among treatments for *S. krylovii* or *A. frigida* grown in mixture

For *S. kryroii*, competition significantly affected biomass accumulation and allocation (all *p* < .05), while immediate nitrogen and water addition only significantly affected some aspects of biomass, especially aboveground biomass. Immediate nitrogen addition significantly increased total biomass by 71.82% (*F* = 13.21, *p* < .001; Table 1, Figure 1). There were significant interactive effects between immediate nitrogen addition and water addition on aboveground biomass and S/R (*F* = 53.78, *p* < .001; *F* = 11.42, *p* < .05; Table 1, Figure 1). Immediate nitrogen addition increased aboveground biomass by 9.20% but decreased S/R by 44.43% without immediate water addition, while it increased aboveground biomass and S/R by 338.89% and 165.05% with immediate water addition, respectively. Immediate water addition suppressed aboveground and S/R by 7.92% and 10.85% without immediate nitrogen addition, but it increased them by 27.01% and 325.25% with immediate nitrogen addition, respectively. Competition effects on total biomass and aboveground biomass were further modified by immediate nitrogen addition (*F* = 5.00, *p* < .05; *F* = 36.45, *p* < .001, N*C, Table 1). Competition increased total biomass by 127.48% or 187.19% without or with immediate nitrogen addition, respectively. Immediate nitrogen addition stimulated total biomass by 45.35% or 83.50% without or with competition was taken into account. Competition increased aboveground biomass by 147.52% or 362.20% without or with immediate nitrogen addition, respectively. Immediate nitrogen addition stimulated aboveground biomass by 276.92% or 348.85% without or with competition, respectively. Similarly, the competition effect on aboveground biomass was further modified by immediate water addition (*F* = 40.86, *p* < .001, Pr*C, Table 1). Competition stimulated aboveground biomass by 109.89% or 427.54% without or with immediate water addition, respectively. Immediate water addition stimulated 17.13% or 194.39% without or with competition, respectively.

**Table 1:** Effects of immediate nitrogen addition (N), immediate increased precipitation (Pr), competition (C), and their interactions on biomass, aboveground biomass, belowground biomass, and S/R of the two species based on three‐way ANOVAs

	*S. kryroii*												*A. frigida*											
	Biomass			Aboveground biomass			Belowground biomass			S/R			Biomass			Aboveground biomass			Belowground biomass			S/R		
	*df*	*F* value	*p* Value	*df*	*F* value	*p* Value	*df*	*F* value	*p* Value	*df*	*F* value	*p* Value	*df*	*F* value	*p* Value	*df*	*F* value	*p* Value	*df*	*F* value	*p* Value	*df*	*F* value	*p* Value
N	1	13.21	<.001	1	60.49	<.001	1	1.94	.17	1	3.29	.08	1	13.22	<.001	1	13.33	<.001	1	8.11	<.05	1	1.91	.17
Pr	1	1.60	.21	1	48.30	<.001	1	1.19	.28	1	8.96	<.05	1	5.19	<.05	1	5.04	<.05	1	3.42	.07	1	0.17	.68
N*Pr	1	3.35	.07	1	53.78	<.001	1	0.32	.58	1	11.42	<.05	1	0.04	.85	1	0.00	.10	1	0.19	.66	1	0.74	.40
C	1	38.09	<.001	1	99.57	<.001	1	12.69	<.05	1	8.81	.05	1	12.67	<.05	1	15.94	<.001	1	4.76	<.05	1	3.94	.05
N*C	1	5.00	.03	1	36.45	<.001	1	0.15	.70	1	2.32	.14	1	2.01	.16	1	3.08	.09	1	0.38	.54	1	1.74	.20
Pr*C	1	3.26	.08	1	40.86	<.001	1	0.06	.81	1	2.19	.15	1	2.60	.12	1	1.98	.17	1	2.48	.12	1	0.64	.43
N*Pr*C	1	5.74	.02	1	48.70	<.001	1	0.05	.82	1	9.54	<.05	1	3.69	.06	1	0.87	.36	1	8.99	<.05	1	6.77	<.05

For *A. frigida*, immediate nitrogen addition significantly increased total biomass, aboveground biomass, and belowground biomass by 103.29%, 129.32%, and 74.42% (*F* = 13.22, *p* < .001; *F* = 13.33, *p* < .001; *F* = 8.11, *p* < .05; Table 1, Figure 1), respectively; immediate water addition significantly increased them by 54.26%, 63.67%, and 42.71% (*F* = 5.19, *p* < .05; *F* = 5.04, *p* < .05; *F* = 3.42, *p* < .05; Table 1, Figure 1), respectively; competition significantly decreased them by 50.00%, 60.09%, and 34.40%, respectively (*F* = 12.67, *p* < .05; *F* = 15.94, *p* < .05; *F* = 4.76, *p* < .05; Table 1, Figure 1). Competition significantly decreased S/R by 28.78% (*F* = 3.94, *p* = .05; Table 1, Figure 1).

**Figure 2:**
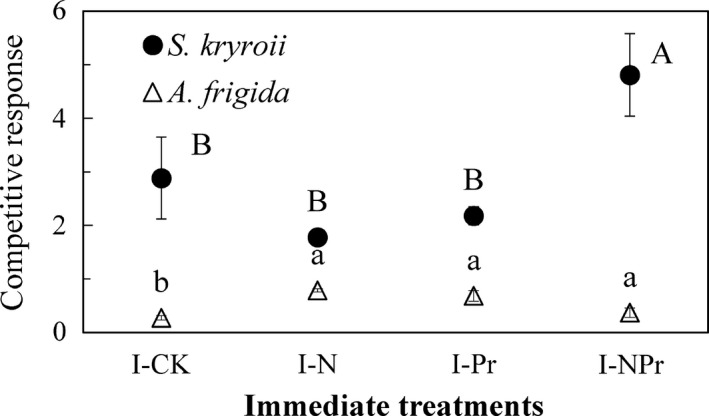
Immediate‐treatment‐induced changes of competitive effects. Values are means ± *SE*, *n* = 6 for each treatment. See Figure 1 for abbreviations. Different uppercase letters indicate significant differences among treatments for *S. krylovii*; different lowercase letters indicate significant differences among treatments for *A. frigida*

Immediate water addition (*F* = 4.40, *p* < .05; Table 2, Figure 2) and its interaction with immediate nitrogen addition (*F* = 11.34, *p* < .05; Table 2, Figure 2) significantly affected the CR of *S. kryroii*. Immediate water addition decreased the CR of *S. kryroii* by 24.42% and increased it by 170.90% without and with immediate nitrogen addition, while immediate nitrogen addition decreased it by 38.42% and increased it by 120.72% without and with immediate water addition, respectively. Immediate nitrogen addition and water addition interacted significantly affected the CR of *A. frigida* (*F* = 34.53, *p* < .001; Table 2, Figure 2). Immediate nitrogen addition increased CR of *A. frigida* by 187.47% and decreased it by 46.04% without and with immediate water addition, respectively; immediate water addition increased it by 149.83% and decreased it by 53.11% under ambient and immediate nitrogen addition, respectively.

**Table 2:** Effects of nitrogen addition, increased precipitation, and their interactions in immediate (I‐N, I‐Pr, I‐N*Pr) and maternal environments (M‐N, M‐Pr, M‐N*Pr) on competitive responses of the two species based on two‐way ANOVAs

	*S. kryroii*			*A. frigida*		
	*df*	*F* value	*p* Value	*df*	*F* value	*p* Value
I‐N	1	1.87	.19	1	2.02	.17
I‐Pr	1	4.40	<.05	1	0.00	.96
I‐N*Pr	1	11.34	<.05	1	34.53	<.001
M‐N	1	5.95	<.05	1	2.11	.16
M‐Pr	1	8.06	<.05	1	1.71	.21
M‐N*Pr	1	7.11	<.05	1	25.84	<.001

### 3.2 Maternal environment affected the species' interspecific relationship

**Figure 3:**
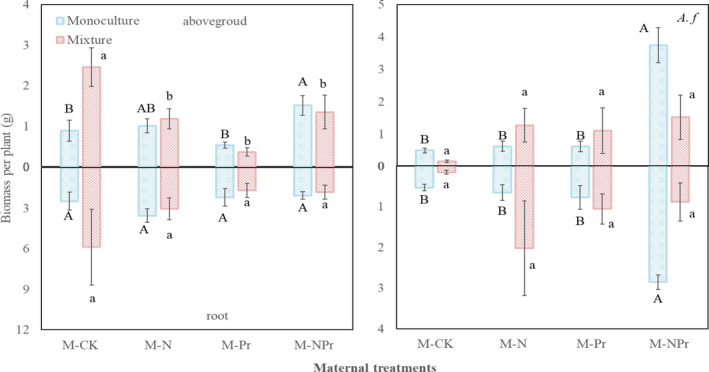
Maternal‐treatment‐induced changes in biomass, aboveground biomass, and belowground biomass of the two species grow in monoculture and in mixture. Values are means ± *SE*, *n* = 6 for each treatment. *S.k*: *Stipa krylovii*, *A.f*: *Artemisia frigida*. M‐CK: control treatment; M‐N: nitrogen addition in Field Experiment (maternal N addition environment); M‐Pr: water addition Field Experiment (maternal‐increased precipitation environment); M‐NPr: nitrogen addition and water addition in combine in field experiment (maternal N addition plus increased precipitation environment). Different uppercase letters indicate significant differences among treatments for *S. krylovii* or *A. frigida* grown in monoculture; different lowercase letters indicate significant differences among treatments for *S. krylovii* or *A. frigida* grown in mixture

For *S. kryroii*, competition and maternal nitrogen addition had no significant effect on biomass and biomass allocation (all *p* > .05, Table 3), while maternal water addition significantly affected biomass accumulation and allocation. Maternal water addition significantly decreased total biomass and belowground biomass by 43.75% and 48.05%, respectively (*F* = 5.70, *p* < .05; *F* = 5.10, *p* < .05; Table 3, Figure 3). There were significant interactions of maternal nitrogen addition and maternal‐increased precipitation on aboveground biomass and S/R (*F* = 15.40, *p* < .001; *F* = 11.35, *p* < .05; Table 3, Figure 3). Maternal nitrogen addition decreased aboveground biomass and S/R by 34.54% and 37.93% without maternal water addition, but it increased them by 218.27% and 131.28% with maternal water addition, respectively; maternal water addition decreased them by 73.14% and 49.89% without maternal nitrogen addition, but increased them by 30.61% and 86.70% with maternal nitrogen addition, respectively. Furthermore, maternal water addition altered the competition effects on aboveground biomass and S/R (*F* = 6.81, *p* < .05, Pr*C, Table 3). Competition stimulated aboveground biomass and S/R by 91.48% and 104.89% without maternal water addition, and it decreased them by 16.33% and 7.43% with maternal water addition, while maternal water addition increased them by 7.77% and 62.17% without competition, and decreased them by 52.91% and 26.73% with competition, respectively.

**Table 3:** Effects of maternal nitrogen addition (N), maternal‐increased precipitation (Pr), competition (C), and their interactions on biomass, aboveground biomass, belowground biomass, and S/R of the two species based on three‐way ANOVAs

	*S. kryroii*												*A. frigida*											
	Biomass			Aboveground biomass			Belowground biomass			S/R			Biomass			Aboveground biomass			Belowground biomass			S/R		
	*df*	*F* value	*p* Value	*df*	*F* value	*p* Value	*df*	*F* value	*p* Value	*df*	*F* value	*p* Value	*df*	*F* value	*p* Value	*df*	*F* value	*p* Value	*df*	*F* value	*p* Value	*df*	*F* value	*p* Value
N	1	0.06	.80	1	1.03	.32	1	0.30	.59	1	0.81	.37	1	12.81	<.001	1	14.28	<.001	1	8.13	<.001	1	3.75	.06
Pr	1	5.70	.02	1	5.02	.03	1	5.10	<.05	1	0.01	.91	1	7.48	<.001	1	12.36	<.05	1	2.57	.12	1	2.18	.15
N*Pr	1	1.68	.20	1	15.40	<.001	1	0.30	.59	1	11.35	<.05	1	0.84	.37	1	3.32	.08	1	0.00	.96	1	5.94	<.05
C	1	0.88	.36	1	3.08	.09	1	0.44	.51	1	2.57	.12	1	0.76	.39	1	1.28	.27	1	0.26	.61	1	1.42	.24
N*C	1	1.76	.19	1	3.07	.09	1	1.27	.27	1	0.79	.38	1	0.86	.36	1	1.88	.18	1	0.14	.71	1	0.17	.66
Pr*C	1	2.33	.14	1	6.81	.01	1	1.33	.26	1	4.10	.05	1	3.78	.06	1	2.62	.11	1	3.82	.06	1	1.75	.19
N*Pr*C	1	2.21	.15	1	3.11	.09	1	1.73	.20	1	1.22	.28	1	10.07	<.05	1	8.51	<.05	1	8.59	<.05	1	4.01	.05

For *A. frigida*, maternal nitrogen addition significantly increased total biomass, aboveground biomass, and belowground biomass by 219.04%, 201.82%, and 155.48% (*F* = 12.81, *p* < .001; *F* = 14.28, *p* < .001; *F* = 8.13, *p* < .05; Table 3, Figure 3), respectively; maternal water addition significantly increased total biomass and aboveground biomass by 112.68% and 175.39% (*F* = 7.48, *p* < .001; *F* = 12.36, *p* < .05; Table 3, Figure 3), respectively. Maternal nitrogen addition and water addition interacted to affect the S/R of *A. frigida* (*F* = 5.94, *p* < .05; Table 3, Figure 3). Maternal nitrogen addition decreased S/R by 8.30% or increased it by 88.50% without or with maternal water addition, while maternal water addition decreased it by 16.31% or stimulated it by 72.03% without or with maternal nitrogen addition, respectively.

**Figure 4:**
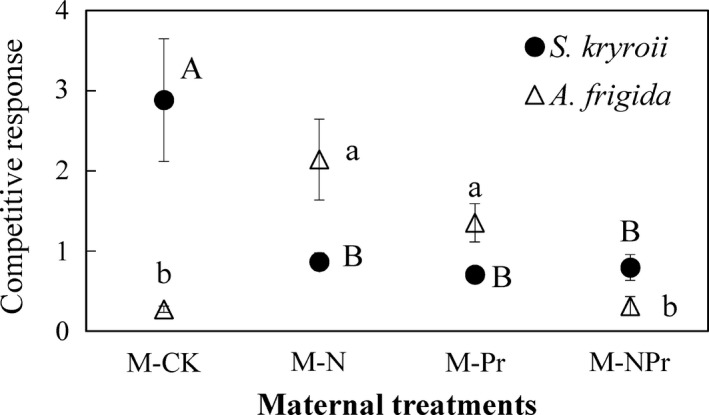
Maternal‐treatment‐induced changes of competitive effect. Values are means ± *SE*, *n* = 6 for each treatment. See Figure 3 for abbreviations. Different uppercase letters indicate significant differences among treatments for *S. krylovii*; different lowercase letters indicate significant differences among treatments for *A. frigida*

Maternal nitrogen addition (*F* = 5.95, *p* < .05; Table 2, Figure 4), maternal water addition (*F* = 8.06, *p* < .05; Table 2, Figure 4), and their interaction (*F* = 7.11, *p* < .05; Table 2, Figure 4) significantly influenced the CR of *S. kryroii*. Maternal nitrogen addition decreased the CR of *S. kryroii* by 69.96% or increased it by 12.63% without or with maternal water addition, while maternal water addition decreased it by 75.52% or 8.23% without or with maternal nitrogen addition, respectively. Although maternal nitrogen addition or water addition had no significant effect (both *p* > .05; Table 2, Figure 4), they interacted to affect the CR of *A. frigida* (*F* = 25.84, *p* < .001; Table 2, Figure 4). Maternal nitrogen addition stimulated CR of *A. frigida* by 684.26% or decreased it by 76.85% without or with maternal water addition, while maternal water addition stimulated it by 394.99% or decreased it by 85.39% without or with maternal nitrogen addition.

## 4 DISCUSSION

### 4.1 Immediate environment affected the species' interspecific relationship

Our results showed that environmental change affected the interspecific competition between the two dominant species. Compared with *A. frigida*, *S. krylovii* exhibited a stronger competitive ability as a whole. Although nitrogen and water added separately decreased the CR of *S. krylovii* and increased the CR of *A. frigida*, *S. krylovii* in mixture benefited from the nitrogen and water addition environments, and it suppressed the accompanying *A. frigida*. Our study showed that nitrogen addition, water addition, and their interaction did change the interspecific competition intensity between the two species, but they did not change the position in the hierarchy (*S. krylovii* out‐competed *A. frigida* under all conditions).

Our results showed that the two dominant species exhibited different strategies to promote competitive ability. In stressful environments, species exhibit phenotypic plasticity in the life history to promote competitive ability, such as alteration of one or a combination of these three fundamental traits: growth, survival, and fecundity (Bolker & Pacala, 1999; Drenovsky et al., 2008; Wang et al., 2010). Furthermore, because of their different intrinsic morphological attributes, plants can choose different strategies to maintain growth, survival, and fecundity (Liu et al., 2016). They may allocate new biomass to leaves and/or roots to increase the plant's capacity to acquire resources (Chapin, 1991; Hermans et al., 2006), or to organs such as stolons, rhizomes, and seeds to increase the ability to exploit new patches (Drenovsky et al., 2008). According to our results, as a gramineous bunch grass, *S. krylovii* significantly stimulated aboveground biomass with nitrogen and water supply in order to intercept light to maintain growth and survival, suppressing *A. frigida* growth in mixture (Niu et al., 2009). For *A. frigida*, although the aboveground and belowground biomass both increased under N addition and increased precipitation, the biomass, especially biomass allocation to shoots, significantly decreased when grown with *S. krylovii*. As a shorter and stoloniferous clonal forb, *A. frigida* increased belowground biomass allocation to capture more resources in soil to maintain growth and survival. Our results were in line with the fact that an inferior competitor could reduce competitive suppression by accessing resources not available to a superior competitor (Carmona et al., 2019). Moreover, although the aboveground biomass of *A. frigida* decreased by 60.09% in the mixture (Figure 1), the largest length of stolon increased by 13.93% in the mixture (Li Y, unpublished data). This means that *A. frigida* in the mixture might allocate more biomass to the stolon for exploring new patches. Based on the size dependence of individual‐level resource use and architecture (Allen et al., 2008), the allometry of biomass partitioning and allocation can explain interspecific competition (Yu & Gao, 2011). According to the nutrition tolerance/resistance and morphology‐related allocation strategy (Yu & Gao, 2011), *S. krylovii* stimulated aboveground biomass to grow higher and increase light interception, while *A. frigida* stimulated belowground biomass to acquire soil nutrients or stimulated stolon biomass to some extent to colonize new patches in our study. Therefore, we considered that *S. krylovii* increased competitive ability through stimulated growth and ability to affect neighbors, whereas *A. frigida* increased competitive ability by stimulating the ability to avoid being affected (Kolodziejek, 2019; Wang et al., 2010).

### 4.2 The maternal environment affected the species' interspecific relationship

Our results are in line with studies which show that the maternal environment may alter offspring performance (Guillaume et al., 2016). However, they were not in line with a previous study showing that perennial plants showed hardly any transgenerational responses (Yin et al., 2019). This is because most of the perennial plants included in that meta‐analysis were trees, whereas those in our study were grass or subshrubs which are more vulnerable to environmental changes (Walter et al., 2016). Moreover, perennial plants may benefit from the transgenerational effect as adaptation is unlikely to keep pace with rapid environmental changes (Herman & Sultan, 2011).

The transgenerational effect of maternal environment on offspring may be attributed to maternal plant phenotypic plasticity to their environment. Species exhibit different phenotypic plasticities to their immediate surroundings via their own physiological optimum (Gilman et al., 2010; Hoffmann & Sgrò, 2011). Thus, the transgenerational effects of the two species in our study were species‐specific, even when maternal plants experienced the same environment. As mentioned above, as a superior competitor, *S. krylovii* invested more nutrients in shoots to acquire light to maintain competitive advantage under favorable immediate conditions. As water addition in this sterile environment only improved limited nutrient availability, the maternal plant of *S. krylovii* might not acquire enough nutrients from the soil to maintain survival, growth, and fecundity (investment in offspring) concurrently, and thus had little nutrient invested in offspring (Li et al., 2019). Thus, offspring biomass may decrease with maternal water addition. However, *A. frigida*, as a weaker competitor, invested equally in biomass for the shoots and the roots to acquire enough soil nutrients under favorable immediate conditions. As the maternal plant of *A. frigida* had accumulated more nutrients to invest in offspring, offspring biomass increased with maternal nitrogen and water addition. Our results were also consistent with previous evidence indicating that lower resource acquisition reduced maternal resource allocation to offspring (Stotz et al., 2018) and that larger plants do not increase offspring performance proportionately (Aarssen, 2015).

Besides the offspring trait, maternal environment affected the offspring competitive ability according to our results and to that of previous studies (Stotz et al., 2018). The effects of the maternal environment and immediate environment may be counteractive (Lau et al., 2008). For example, according to our results, unlike the immediate environment's effect, the transgenerational effect of maternal environment decreased *S. krylovii*'s competitive ability and increased *A. frigida*'s competitive ability to some extent. The maternal environment significantly decreased the biomass of *S. krylovii* offspring and increased that of *A. frigida* in mixture, and it may be responsible for such a competitive ability change. Overall, the transgenerational effect should lead to a decline in *S. krylovii* in its dominance induced by the immediate environment over time. On the contrary, the transgenerational effect should relieve the competitive suppression of *A. frigida* induced by the immediate environment. Thus, the transgenerational effect should further facilitate the coexistence of the two species under scenarios of nitrogen addition and increased precipitation, as it would result in no species being stronger or weaker.

The specific transgenerational effect on the competitive ability of the two species may be attributed to different population development strategies. In order to maximize population fitness, annual plants improve population fitness mainly by increasing investment to offspring (Neytcheva & Aarssen, 2008), whereas perennial plants have more tradeoffs (Yin et al., 2019). To avert the deleterious consequences of environmental change, immediate action must be taken for the survival and growth of perennial plants (Auge et al., 2017; Suzuki & Teranishi, 2005; Tracey & Aarssen, 2011, 2014), and strategic conservation planning for the coming years and offspring is necessary (Dawson et al., 2011). That is, plant populations exhibit phenotypic plasticity to cope with environmental changes either in the generation or in filial generations (Holeski et al., 2012; Van Dam & Baldwin, 2001). There are no general apparent advantageous strategies for population development. For example, if the environment fluctuates at a high frequency, within‐generational plasticity will be advantageous for superior competitors or species with rapid plasticity growth (Auge et al., 2017; Kuijper & Hoyle, 2015), while weaker competitors relieve competitive suppression through avoidance (Cahill et al., 2010), which allocate more resources to offspring instead of growth (Aarssen, 2015). Thus, although the direction and magnitude may be different between the maternal environment effect and immediate environment effect for the two species in our study, we cannot determine whether the transgenerational effect is adaptive based on few offspring traits, especially for perennial plants. As a plant population consists of individuals of different generations, assessing the adaptive value of transgenerational effect on one generation's fitness is insufficient (Auge et al., 2017; Uller, 2008). For the purpose of maximizing population fitness, the effective transgenerational effect within a given habitat should be defined of the several alternative combinations of traits of the maternal plant and offspring plant (Bittebiere et al., 2019; Moles, 2018). According to our results, *S. krylovii*, as a superior competitor, takes advantage of a favorable immediate environment to promote competitive ability because the significant and positive effects of immediate N addition, water addition, and their interaction on competitive response and CR are higher than those of *A. frigida*. The maternal environment had a significant and negative effect on the CR of *S. krylovii* and a significant and positive effect of the interaction between maternal N and water addition on the CR of *A. frigida*. Therefore, *A. frigida*, as a weaker competitor, may partly benefit from maternal‐favorable environment to promote competitive ability over time. This is consistent with previous studies showing that species adapt to environmental change via immediate effect or transgenerational effect depending on the limit and cost of within‐ or across‐generation plasticity (Auge et al., 2017; Kuijper & Hoyle, 2015). Our results also imply that competitive success is defined not only by the capacity to capture resources and deny them to neighbors, but also by the capacity to withstand suppression from competition enough to persist, both as an individual and across generations.

### 4.3 The interaction of N addition and increased precipitation

Water and nitrogen are the most frequent limiting resources in temperature steppes (Xu et al., 2018). Changes in nitrogen and water availability are known to influence vegetation dynamics and ecosystem processes (Li et al., 2011). Water is essential for nutrient diffusion and replenishment in soil (Zhang et al., 2015). High water availability may contribute to the replenishment of added N to the soil solution and consequently increase available nitrogen for plant growth. Thus, in arid and sterile environments, water and nitrogen addition usually play a significant interaction. As a superior competitor, *S. krylovii* used the increase in nitrogen availability to stimulate its biomass in the mixture in an immediate nitrogen and water coinstantaneous added environment. This may explain the significant interaction between nitrogen addition and water addition in the competitive responses of *S. krylovii* in our study. However, *S. krylovii* could not transfer competitive advantage to its filial generation because it took advantage of favorable immediate environments to promote competitive ability. Although the maternal nitrogen and water coinstantaneous added environment substantially stimulated the biomass of *A. frigida* in the monoculture, as a weaker competitor, *A. frigida* in the mixture stimulated little biomass and consequently exhibited a lower CR. The interaction of water addition and nitrogen addition to arid and sterile environments showed that *S. krylovii* stimulated competitive ability in the generation and *A. frigida* stimulated biomass accumulation in the filial generation to some extent. Therefore, it is more important to take the transgenerational effect on the interspecific competition of offspring into account under future increased precipitation and nitrogen addition.

## 5 CONCLUSIONS

Our results suggest that species chose one of a large number of strategies to become superior competitors or to avoid competitive suppression in a community under environmental change. The plant population exhibited phenotypic plasticity to cope with environmental changes either in the generation or in the filial generations. Population development strategies affected offspring interspecific relationships through transgenerational effects. Moreover, our results on the maternal environment's transgenerational effect on the two dominant species' growth and relationship demonstrated that the community composition of this steppe may be misestimated if we depend on the immediate environmental effect on interspecific competition, this is because of the overestimation of *S. krylovii*'s competitive advantage and population development and the underestimation of *A. frigida*'s competitive advantage and population development. In conclusion, our results demonstrated that the transgenerational effect of maternal environment on offspring interspecific competition must be considered when predicting population dynamics and community composition under global change scenarios.

## CONFLICT OF INTEREST

The authors declare that they have no conflict of interest.

## AUTHOR CONTRIBUTIONS

**Yang Li:** Conceptualization (equal); Data curation (equal); Formal analysis (lead); Methodology (equal); Writing‐original draft (lead); Writing‐review & editing (equal). **Longyu Hou:** Conceptualization (supporting); Data curation (supporting); Formal analysis (supporting); Methodology (supporting); Writing‐original draft (supporting); Writing‐review & editing (supporting). **Liuyi Yang:** Conceptualization (supporting); Data curation (supporting); Formal analysis (supporting); Methodology (supporting); Writing‐original draft (supporting); Writing‐review & editing (supporting). **Ming Yue:** Conceptualization (equal); Data curation (equal); Formal analysis (equal); Methodology (equal); Writing‐original draft (equal); Writing‐review & editing (equal).

## Data Availability

The data that support the findings of this study are openly available in the Dryad Digital Repository at https://doi.org/10.5061/dryad.n8pk0p2sk
